# Supplementary material for: Increased Heparanase Levels in Urine during Acute Puumala Orthohantavirus Infection Are Associated with Disease Severity
Source: Viruses. 2022 Feb 22;14(3):450. doi: 10.3390/v14030450 (PMC8954369; doi:10.3390/v14030450)
Supplement: Supplementary file 1 [file viruses-14-00450-s001.zip › viruses-1583532-supplementary.pdf]

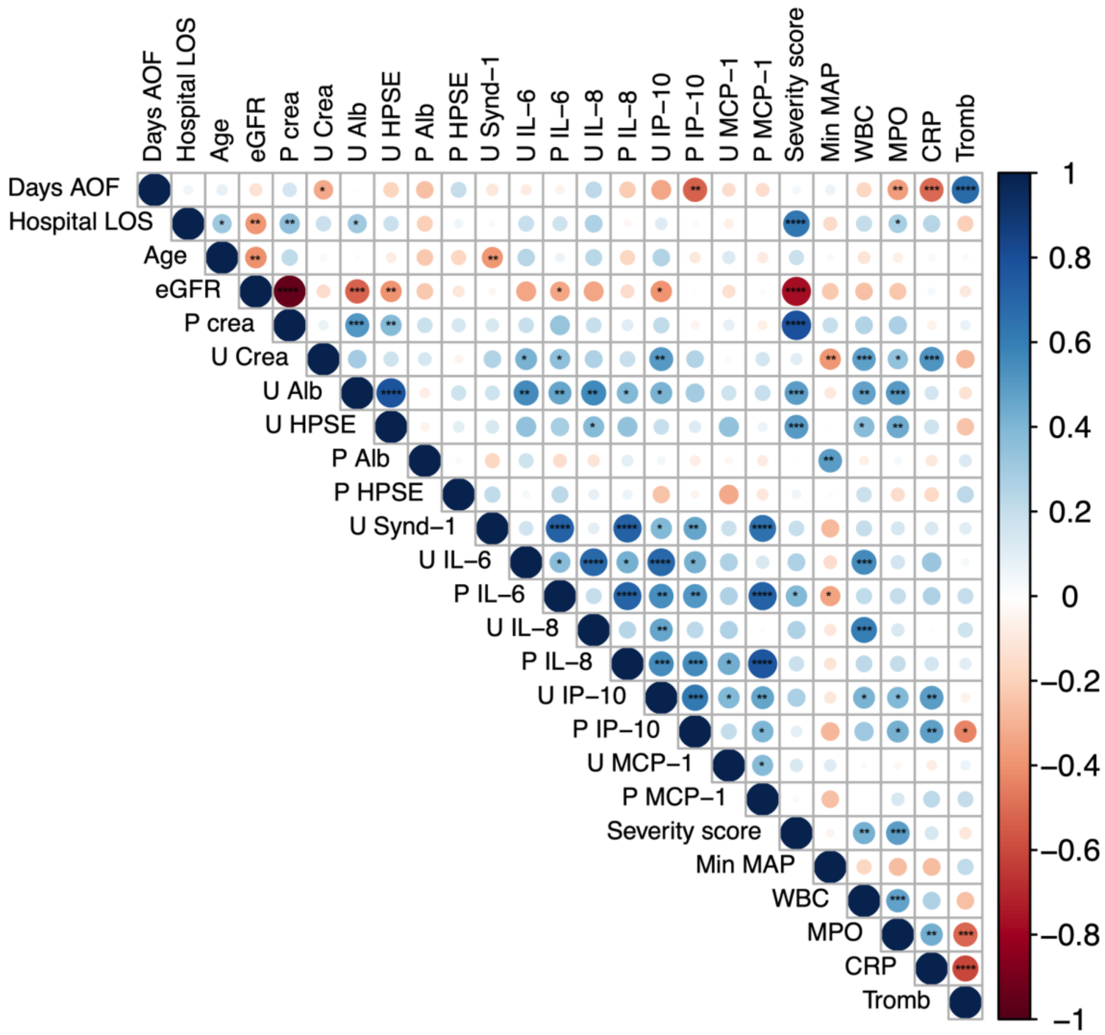

Figure S1. Spearman rank correlation coefficient matrix using absolute concentrations of variables measured from urine.
